# Microbial diversity and fitness in the gut–brain axis: influences on developmental risk for Alzheimer’s disease

**DOI:** 10.1080/19490976.2025.2486518

**Published:** 2025-04-10

**Authors:** Angelo M. Jamerlan, Seong Soo A. An, John P. Hulme

**Affiliations:** Department of Bionanotechnology, Bionano Research Institute, Gachon University, Seongnam-si, Republic of Korea

**Keywords:** Alzheimer’s disease, gut-brain axis, quorum sensing, microbiota-gut-brain axis, neuromodulators, microbial diversity, gut dysbiosis, neurodegenerative diseases, probiotics, neuroinflammation

## Abstract

The gut–brain axis (GBA) denotes the dynamic and bidirectional communication system that connects the gastrointestinal tract and the central nervous system (CNS). This review explored this axis, focusing on the role of microbial diversity and fitness in maintaining gastrointestinal health and preventing neurodegeneration, particularly in Alzheimer’s disease (AD). Gut dysbiosis, characterized by the imbalance in populations of beneficial and harmful bacteria, has been associated with increased systemic inflammation, neuroinflammation, and the progression of AD through pathogenic mechanisms involving amyloid deposition, tauopathy, and increased blood–brain barrier (BBB) permeability. Emerging evidence highlighted the therapeutic potential of probiotics, dietary interventions, and intermittent fasting in restoring microbial balance, reducing inflammation, and minimizing neurodegenerative risks. Probiotics and synbiotics are promising in helping improve cognitive function and metabolic health, while dietary patterns like the Mediterranean diet were linked to decreased neuroinflammation and enhanced gut–brain communication. Despite significant advancement, further research is needed to elucidate the specific microbial strains, metabolites, and mechanisms influencing brain health. Future studies employing longitudinal designs and advanced omics technologies are essential to developing targeted microbiome-based therapies for managing AD-related disorders.

## 1. Introduction

The human body contains 100 trillion bacteria, 80% of which are found in the GI tract.^1^ Over 100 bacterial species encode 150 times more genes than the human genome.^2^ The gut environment plays various crucial roles, such as creating an intestinal barrier by producing mucus, supporting the sustained presence of the gut microbiota, encouraging the renewal of epithelial cells, and generating short-chain fatty acids (SCFAs) that nourish the mucosa.^1^ The gut microbiota, composed predominantly of Bacteroidetes and Firmicutes, plays a central role in maintaining gastrointestinal and systemic health. Alongside smaller populations of Proteobacteria, Actinomyces, Fusobacterium, and Verrucomicrobia, these microbes form a dynamic ecosystem critical to host function.^1,3^ Different bacterial populations also communicate to regulate their environment and maintain the optimal health of the host. They coordinate their gene expression and behavioral processes in a cell density-dependent manner through quorum sensing (QS).^4^ Chemical signaling compounds produced and released by bacteria during QS are called quorum-sensing molecules (QSMs).^5^

**Figure 1.**
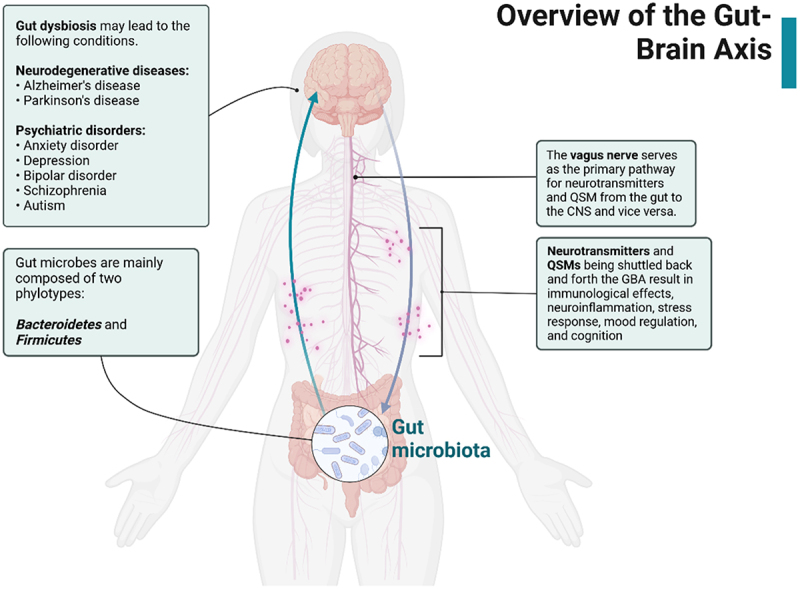
Overview of the gut–brain axis. Gut microbes release neurotransmitters and QSMs that travel through the vagus nerve and influence the CNS. In the event of gut dysbiosis, neurodegenerative disorders may arise as a result of the stress response, immunological response, and neuroinflammation. Created with BioRender.com.

The gut microbiota also extends its communication to host cells to maintain gut homeostasis. This was evidenced by numerous studies that altered or eliminated the microbiota from the GI tract through germ-free animal models, producing abnormal gastric motility, neurodevelopmental defects, and a dysregulated immune system.^12,13^ The gut microbiota and the GBA are collectively called the microbiota‒gut‒brain axis (MGBA). However, there are instances when various factors perturb this delicate balance in microbial populations, and the growth of microbes that increase systemic inflammation and gastrointestinal permeability is favored, increasing the risk of AD pathogenesis.^14–16^ Gut dysbiosis also results from bacterial amyloids that promote the growth of harmful bacteria and can also permeate weakened or leaky barriers due to aging, chronic inflammation, and other comorbidities, accelerating AD progression.^17^ Building on these molecular pathways, interventions like dietary modulation and targeted microbial therapies present actionable approaches to restore microbial homeostasis. These strategies address gastrointestinal health and hold promise in mitigating neurodegenerative risks associated with MGBA dysregulation (Figure 1). This review discusses the impact of decreased gut microbial diversity, its connection with Alzheimer’s disease pathogenesis, related pathogenic risk factors, and possible dietary interventions that may aid in reducing the likelihood of developing the disease.

## 2. The gut–brain connection

Disruptions in gut microbial balance can profoundly affect host physiology, mainly through the microbiota–gut–brain axis (MGBA). This intricate network connects the central nervous system (CNS), enteric nervous system (ENS), digestive cells (enterocytes), and gut microbiota, mediating bidirectional communication.^1,18^ Enterocytes and the neurons of the ENS form the interface that separates the gut microbiota and the CNS. In between, various peptides and amines are produced that influence this communication.^19^ The exact molecular pathways that govern this complex network are still being elucidated, but several animal studies have uncovered the effects of differences in gut microbial communities on brain function.

Germ-free mice models raised in sterile laboratory conditions that made them devoid of any microbial life revealed the valuable role of the gut microbiota in stress responsivity, anxiety-like behaviors, sociability, and cognition.^12^ Early colonization patterns and critical developmental windows also influenced behavioral outcomes and cognition.^20^ A study recently showed that heavy metal exposure could modify gut microbiota composition, potentially impacting brain function and behavior.^21^ Subdiaphragmatic vagotomy on mice prevented anxiolytic effects and immune changes resulting from *Lactobacillus rhamnosus* administration, underscoring the valuable role of the vagus nerve in microbiota–gut–brain signaling.^22^ Vagotomy also positively impacted fecal microbiota transplantation and behavioral and biochemical alterations induced by stress in rats.^23^ Hepatic branch vagotomy in cirrhotic mice modified the gut microbiome, liver, and brain inflammation, indicating its potential involvement in regulating the gut-liver-brain connection.^24^ Specific signaling also enabled the vagus nerve to identify whether bacteria were pathogenic, with the potential for anxiogenic and anxiolytic effects to be induced, depending on the stimulus.^25^ Dysbiosis and vagal dysfunction were also associated with various GI and psychiatric disorders, including inflammatory bowel disease (IBS).^26^

The vagus nerve was revealed as a principal conduit between the ENS and the CNS, sensing microbial metabolites and transmitting information from the gut to the brain.^26^ However, it should be noted that several other connections and mechanisms influence the bidirectional communication between the gut and the brain. Stress activates the hypothalamic–pituitary–adrenal (HPA) axis, causing the hypothalamus to release corticotropin-releasing factor (CRF). This stimulates the pituitary gland to secrete adrenocorticotropic hormone (ACTH), releasing cortisol from the adrenal glands. Cortisol is an important stress hormone that helps regulate the body’s use of fats, proteins, and carbohydrates during the fight-or-flight response. It also assists in maintaining healthy glucose levels in the blood by promoting gluconeogenesis in the liver. This increases the availability of energy needed while suppressing non-essential functions. The gut is also lined with enteroendocrine cells that release peptides and hormones (e.g., ghrelin, galanin) that stimulate appetite by acting on the hypothalamus and influencing mood, feeding behavior, and pain.^18^ The autonomic nervous system (ANS) also contributes to bidirectional signaling and similarly influences gut motility, secretion, and immune responses.^18^ The connections between the gut and the brain are numerous and complex, and the data showed that the state of the gut microbiome heavily influenced the integrated gut and brain functions.

## 3. Quorum sensing in the gut

As a critical communication mechanism, quorum sensing (QS) enables bacterial populations to coordinate collective behaviors in response to environmental cues. This process begins with the production of AIs, which are released into the extracellular environment through passive diffusion. As the population density of the bacteria increases, the AI concentration increases accordingly. Once the critical concentration is reached, the release of AIs from the cells becomes energetically unfavorable, increasing their intracellular concentrations. This promotes AI binding with specific receptor proteins, triggering signaling cascades and altering transcription factor activity and gene expression.

QS has various effects on bacterial populations, including modulating gene expression, facilitating communication, altering virulence factors, promoting or hampering biofilm production, and interfering with the growth of other microorganisms.^27,28^ This interference with the development of other microorganisms through quorum-quenching enzymes and antagonistic autoinducer molecules was notable. It highlighted the intricacies of microbes and dynamic communication networks, which employed various strategies to gain a competitive edge within their communities.^29^ The commonly studied QSMs are summarized in Table A1 in the Appendix.

In the gut, QS helps bacteria adapt to the dynamic environment by regulating collective behaviors essential for survival and colonization. For instance, QS could control biofilm formation, providing a protective environment for bacteria and increasing their resistance to external stresses.^5^ Moreover, QS influences the production of antimicrobial compounds and other public goods, which are molecular resources released into the extracellular environment that benefit the entire bacterial community.^30^ The intricate regulation of these resources through QS helps maintain a balanced microbial community, preventing the overgrowth of any single species while promoting a stable and diverse microbiome.^5^ This homeostasis is essential for gut health, supporting digestion, nutrient absorption, and protection against pathogenic bacteria. QS also influences the production of toxins, enzymes, and other QSMs that contribute to the bacterium’s pathogenicity. For instance, in *Pseudomonas aeruginosa*, QS was observed to regulate the production of pyocyanin, elastase, and rhamnolipids, which were critical for tissue damage and immune evasion.^31^ Biofilm production was another process that helped enhance antibiotic resistance and immune responses, making infections more difficult to treat.^32^ QS allowed bacteria to synchronize their activities, ensuring that virulence factors were produced only when the bacterial population reached a critical density.^33^ This intricate coordination enabled pathogenic bacteria to mount a united attack, effectively overwhelming the host’s numerous immune defenses, rather than acting individually.^34^ The expression of factors aiding bacterial evasion of the immune system occur through QS by disrupting immune signaling and degrading specific immune components. Certain QSMs also modulate host cell signaling pathways, producing altered immune responses and exacerbating tissue damage.^33^ Antibiotic resistance genes could also be shared between bacteria through horizontal gene transfer, allowing bacterial resistance to spread across the population.^35^

## 4. Bacterial endocrinology and host interactions

While QS mainly involves communication between bacterial communities to coordinate their collective behavior, bacteria also release hormones and neuromodulators that can affect host cells.^36,37^ Hormones are thought to stimulate the host’s neurophysiology through two main routes: (1) direct interaction, where they bind to the specific receptors in the GI tract, and (2) absorption and circulation, where they are absorbed through the GI wall, enter the portal circulation, and eventually reach the brain.^38^ One striking aspect of the microbiota–gut–brain axis (MGBA) is the role of bacterial synthesis of neurotransmitters. Microbial production of serotonin, dopamine, and gamma-aminobutyric acid (GABA) directly impacts the central nervous system (CNS), influencing mood, social cognition, and interaction.^39^ This was supported by studies demonstrating that decreased gut microbial diversity was linked to depression and anxiety disorders. The deficiency of specific beneficial bacteria contributes to the body’s impaired capacity to synthesize essential hormones.^40–42^ Other molecules, such as SCFAs, were indirectly synthesized through the fermentation of dietary fibers and influenced the host’s metabolism and immune response.^43^

**Figure 2.**
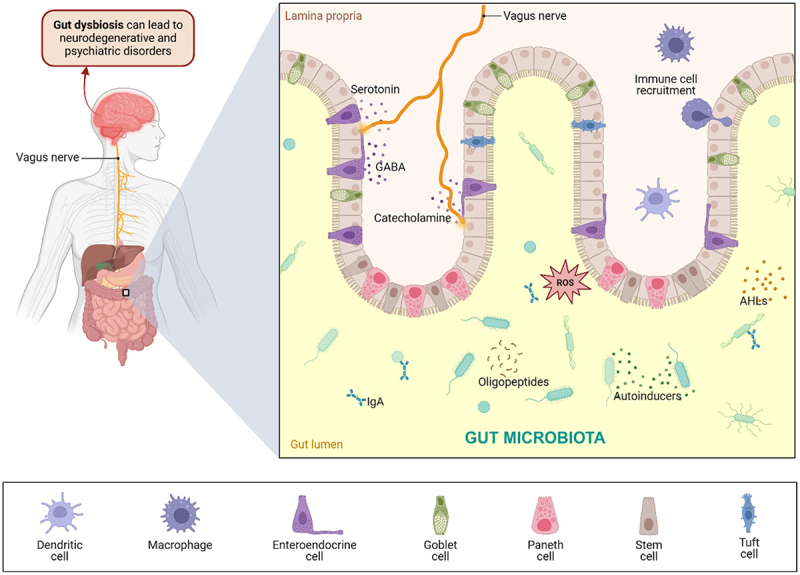
Cross-kingdom signaling in the GBA. The gut microbiota releases several neuromodulators, such as neurotransmitters, oligopeptides, and other metabolites, interacting with epithelial and immune cells. Specialized cells lining the intestinal epithelium maintain intestinal homeostasis, form a mucus barrier, and facilitate the passage of material through the intestines.^45^ neurons also recognize neurotransmitters, which travel through the vagus nerve to the brain. Gut dysbiosis resulting from imbalanced gut microbial populations leads to immunological effects and neuroinflammation, possibly resulting in neurodegenerative and psychiatric disorders. Created with BioRender.com.

Changes in gut microbiota composition could influence dopaminergic signaling, which impacts behaviors related to reward and motivation.^44^ In addition to AHLs, SCFAs, and neurotransmitters (GABA, serotonin, dopamine), other neuromodulators, such as tryptophan metabolites, peptidoglycan, lipopolysaccharides (LPS), and oxylipins, are also involved in gut microbial–host interactions and have several downstream effects on immune response, fluctuations in gut microbial diversity, mood changes, and cognitive functions (Figure 2).^46,47^ The neuromodulators are initially produced in the gut by bacteria and, while in the lumen, interact with the ENS, which is a neuronal network embedded in the gut wall. The ENS can function independently but also communicates with the CNS through neuroendocrine and metabolic pathways.^18^ The neuromodulators then activate sensory afferent neurons in the gut, and the signals are relayed from the gut to the brain by traveling through the vagus nerve. Other neuromodulators and metabolites could enter circulation, penetrate the blood–brain barrier (BBB), and directly influence brain function. For instance, SCFAs affect brain activity by modulating the sympathetic nervous system and influencing neurotransmitter levels.^48^

## 5. Gut microbial diversity and dysbiosis

**Figure 3.**
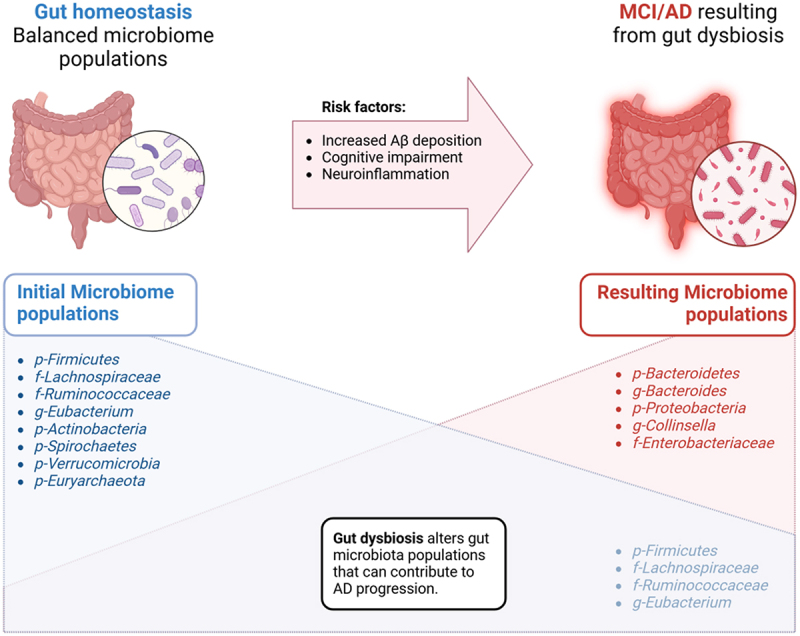
Dynamic alterations in the GBM. Shifts in different microbiota populations resulting from gut dysbiosis can enhance present risk factors that increase the likelihood of developing MCI/AD pathology. Created with BioRender.com.

Understanding gut microbiota diversity and its health implications has been significantly advanced by sequencing technologies. These tools, such as 16S rRNA analysis and metagenomics, have provided valuable insights into microbial community composition and their functional roles. Large-scale projects such as the NIH-Human Microbiome Project have revealed using shotgun metagenomics that the most abundant bacteria phyla in the human gut are Bacteroidetes and Firmicutes.^49^ A healthy balance of different microbial populations indicated the decreased likelihood of CNS-related disorders and inflammatory bowel disease (IBD) (Figure 3).^50–52^ Building on the knowledge that gut microbiota is significantly associated with host health, Gupta et al. developed the Gut Microbiome Health Index (GMHI), an algorithm designed to predict the likelihood of disease independently of clinical diagnosis. Fifty beneficial microbial species were identified from the analysis of 4,347 human stool metagenomes and aided in calculating the GMHI.^53^ The same group published an improved index (GMHI 2) by pooling 8,069 stool shotgun metagenomes from 54 published studies globally to differentiate pathogenic gut taxonomic signals.^54^

Clinical studies and pre-clinical models have been conducted to better understand the relationship between alterations in gut microbial populations and AD development. Table 1 summarizes these studies utilizing mouse models and investigations that analyzed fecal samples from AD patients to identify differences in gut microbiota composition compared to healthy controls.

**Table 1.** Summary of preclinical and clinical studies highlighting gut microbiota alterations in AD.

Study Type	Key Findings	Microbial Contributions to AD	References
Preclinical	Increased Firmicutes and Proteobacteria in aging WT and AD mice; Verrucomicrobia significantly decreased in old AD mice.	Reduction of SCFAs, i.e., butyric acid, was associated with amyloid deposition and ultrastructural abnormalities in AD mice.	Zhang et al.^6^
	Increased abundance of these bacteria in AD mice compared to WT littermates following Aβ exposure.	Possible triggering of adaptive response Altered gut environment that favors microbial growth Altered interaction between host immune system and gut microbiota in AD.	dos Santos Guilherme et al.^7^
	Gut microbiota transplantation from AD mice induced behavioral changes and accelerated cognitive impairment in healthy recipients.	Tau phosphorylation is impacted by the butyric acid-mediated acetylation of GSK3β at lysine 15, regulating the phosphorylation at serine 9.	Zhang and Shen et al.^8^
	Fecal microbiota transplantation (FMT) from healthy mice reduced amyloid plaques and NFTs, improved cognitive function, and restored gut microbiome diversity	Long-term transfer of healthy fecal microbiota to ADLP^APT^ mice restored intestinal macrophage activity and circulating blood Ly6Chi monocytes, as similar levels to those of WT mice.	Kim et al.^9^
Clinical	16S rRNA sequencing of fecal samples from AD patients showed reduced Firmicutes and increased Actinobacteria and Verrucomicrobia compared to healthy controls.	Shift from butyrate-producing bacteria to lactate producers contributes to immune disturbances in the host. Bacteria could utilize altered biosynthesis and metabolism of fatty acids in AD to produce immunomodulatory metabolites	Ling et al.^10^
	Increased Bacteroidetes and decreased Actinobacteria in AD patients	Increased permeability and inflammation is potentially associated with higher risk for AD	Zhuang et al.^11^

Moreover, plant-based foods were beneficial in increasing gut microbial diversity.^55^ Compounds such as SCFAs, plant fibers, and chitosan were linked to increased beneficial bacterial populations in the developing gut.^56–59^

Though often understated, the unique anatomy of the gut also plays a crucial role in maintaining microbial homeostasis, potentially influencing the risk for dysbiosis. Each section of the digestive tract has a distinct environment that influences the growth of essential microbes, potentially leading to dysbiosis when these conditions are significantly altered. For instance, the small intestine is more acidic, having higher levels of oxygen and antimicrobials and shorter transit time, thus favoring the growth of fast-growing facultative anaerobes like Lactobacillaceae and Enterobacteriaceae.^60^ Specialized epithelial immune cells called Paneth cells at the base of the crypts of the small intestine secrete various antimicrobial peptides that restrict bacterial growth near the mucosal surface.^61^ The proximal end of the small intestine also contains a higher concentration of these antimicrobials, resulting in a higher abundance and diversity of bacteria in distal locations.^60^

Compared with other sections in the gut, the cecum was known to contain a distinct microbial community. Studies revealed a greater diversity of bacteria, including Ruminococcaceae and Lachnospiraceae, which are less prevalent in the colon.^60,62^ Different bacterial populations in these nodes also produced unique metabolites that impacted the ENS and, consequently, the CNS, affecting mood and cognitive functions.^18^ The lower concentrations of antimicrobials in the cecum and colon, along with the slower transit time and limited availability of simple carbon sources, promote the growth of fermentative, polysaccharide-degrading anaerobes. This includes high-abundance families such as Bacteroidaceae and Clostridiaceae.^60^ The increased plethora of bacteria requires these sections, particularly the colon, to have a thicker mucus barrier, shielding vulnerable epithelial cells from direct bacterial contact. This barrier consists of two distinct layers: an outer layer that supports mucin-degrading bacteria, such as *Bacteroides acidifaciens* and *Bacteroides fragilis*, and an inner layer that fosters a more restricted microbial community, including *Bacteroides fragilis* and *Acinetobacter spp*.^60^ Due to their depth, colon crypts can harbor unique microbial communities and, more importantly, become reservoirs for bacteria, maintaining stability and supporting recovery after perturbations.^60^ Diet, the use of antibiotics, activation of the immune system, and the physiological changes associated with aging are all crucial factors that can influence the diversity of the gut microbiota.^63–66^ These factors may contribute to gut dysbiosis, which could lead to neuropathological effects, including Alzheimer’s disease.

## 6. Effects on Alzheimer’s disease pathogenesis

Alzheimer’s disease (AD) is a neurodegenerative disorder that leads to severe memory and cognitive impairment. This disease is caused by the progressive deposition of amyloid and tau proteins, which form plaques and neurofibrillary tangles in cortical tissue.^67,68^ Neuronal connections are disrupted by these deposits, eventually leading to cell death.^67,68^ Several factors contribute to the pathogenesis of the disease, including the unregulated activity of gamma and beta secretases, which cleave Aβ oligomers from amyloid precursor protein (APP), a transmembrane protein, and the abnormal clearance of Aβ and tau.^69^

The staging of AD from a neuropathological perspective is determined by the specific brain regions affected by the pathology. The severity of the pathology is evaluated using a semi-quantitative approach.^70^ The models use an ABC scoring system that designates stages to amyloid-β plaques (A), the Braak stage of tau neurofibrillary tangles (B), and the CERAD (Consortium to Establish a Registry for Alzheimer’s Disease) score for neuritic plaques (C).^71,72^ The spread of amyloid plaques is staged according to Thal staging, which describes the initial stage as the deposition of Aβ across the whole neocortex (phase 1), followed by the isocortex (entorhinal and hippocampal cortices, phase 2), the striatum and diencephalon (phase 3), brainstem nuclei (phase 4), and the cerebellum (phase 5).^73^ Braak and Braak developed the staging of tau neurofibrillary tangles. It begins in the transentorhinal cortex (stage I), followed by the involvement of the entorhinal cortex and hippocampus (stage II). Next, it affects the inferior temporal neocortex (stage III) and then progresses to the association cortices (stages IV and V) and the primary sensory cortices (stage VI).^74–76^ Therriault et al. have pointed out in their recent review of the status of AD staging the importance of considering different brain regions of interest (ROI) when assessing tau pathology. This is due to the high variability of tau deposition and topography that is associated with AD symptoms and neurodegeneration. By focusing on areas like the transentorhinal cortex, entorhinal cortex, and hippocampus, where tau tangles typically begin to accumulate, the diagnostic correlation of tau pathology in asymptomatic adults is significantly improved.^77^

Amyloid concentrations in CSF were positively correlated with the abundance of *Alistipes spp*., *Blautia spp*., *Bacteroides spp*., and *Odoribacter splanchnicus*. In contrast, Bacillota showed a positive correlation with Aβ concentrations in plasma. Conversely, Aβ CSF concentrations were negatively correlated with Erysipelotrichaceae and *Lactobacillus spp*.^11,78–81^ Cattaneo et al. found that amyloid-positive patients exhibited elevated levels of proinflammatory cytokines, such as interleukin (IL)-6, CXCL2, NLRP3, and IL-1B, in contrast to the lower levels of the anti-inflammatory cytokine IL-10.^78^ Bacterial amyloids can also trigger cross-seeding of misfolding proteins through molecular mimicry.^17,82^

While the deposition of Aβ is a hallmark of AD, their presence does not always correlate significantly with the severity of cognitive impairment.^83–85^ As the tauopathy spreads due to the formation of neurofibrillary tangles, the relative pathologic significance of Aβ significantly declines.^86^ Gut dysbiosis resulting from dramatic changes in microbiota population profiles is typically characterized by increased inflammation, plaque formation, and neurotransmitter alterations.^87^ Focusing on tau pathogenesis leading to AD in this context is just as relevant, as pre-tangle tau initiates AD pathogenesis.^88^ Recently, several mechanisms that potentially link gut dysbiosis with tauopathy in AD were summarized by Zhu et al. in a review. Three studies in AD cohorts across the globe consistently reported a decreased relative abundance of Firmicutes and an increased abundance of Bacteroidetes.^11,79,89,90^ However, the ratio of Firmicutes to Bacteroidetes was not explicitly stated.^90^ Five of the seven clinical studies also utilized 16S rRNA gene amplicon sequencing (V3-V4 variable region) rather than shotgun metagenomic sequencing.^90^ Since the V3-V4 variable region constitutes only a tiny part of the entire bacterial genome, metagenomic sequencing is necessary to identify microbial strains and structural variations unique to patients with tauopathy – nevertheless, the mechanisms describing how the gut microbiota directly impact tauopathy are still unclear. As noted by Zhu et al., it is crucial to investigate if there are any CNS-associated microbiota that can potentially translocate through enteric nerve endings in patients with tauopathy.^90^ Investigating the roles of microbial metabolites in modulating microglial morphology, energy metabolism, and expression of inflammatory genes in response to pathogenic insult are just as important. As both T cells and microglia exacerbate tau-mediated neurodegeneration, the role of metabolites in influencing innate and adaptive immunity in tauopathy is also an interesting avenue.^90^

Finally, AD is classified into two types depending on the age of onset: (1) Early-onset AD (EOAD), which occurs in people younger than 65 and mostly in their 40s or 50s, and (2) Late-onset AD (LOAD), which typically develops after 65.^91,92^ EOAD is primarily caused by mutations in three genes: APP, PSEN1, and PSEN2.^93^ It is the rarer form, and estimates of its prevalence range from 0–1%^94^ to 6–7%^95^ of all AD cases. In contrast, LOAD is more common and involves more risk factors besides genetic predisposition, which include epigenetics and environmental factors.^93^ While mutations in APOE, PICALM, BIN1, and TREM2 were identified,^96^ environmental factors also significantly influence LOAD development. Among these risks are occupational exposures, preexisting medical conditions, and lifestyle factors such as exercise, education, and nutrition.^97,98^ Age is the most significant risk factor, significantly increasing the risk of LOAD, especially impacting 65 out of 100 people aged 80 and older.^99^ Thus, we can assert that gut dysbiosis and its downstream effects, such as inflammation, significantly contribute to the increased risk of developing AD, particularly LOAD, whose etiology is more complex due to the multitude of interacting risk factors. Thus, the impact of gut dysbiosis was observed to contribute significantly to the etiology of AD.

### 6.1. Inflammation, leaky barriers, and oxidative stress drive AD pathogenesis

Neuroinflammation is a critical factor in the progression of Alzheimer’s disease (AD), significantly influencing its development and symptoms.^100^ The MGBA plays a crucial role in this process,^14–16^ with the gut microbiota influencing brain function through specific metabolites.^8,101^ Gut microbial populations were mainly divided into pro-inflammatory GM, considered drivers of inflammation, and anti-inflammatory GM, which helped restore gut homeostasis.^102^ These bacteria release specific metabolites that influence inflammation in various ways.^103,104^ Gram-negative bacteria, for instance, produce LPS that can bind to toll-like receptor 4 (TLR4) on immune cells that activate them.^105^ Specific pro-inflammatory cytokines such as tumor necrosis factor α (TNF- α) and IL-6 are also produced and were considered key drivers of inflammation.^106^ Anti-inflammatory bacteria mitigated the effects of pro-inflammatory species by producing SCFAs like butyrate. Haran et al., using metagenomic sequencing, revealed relatively lower abundances of butyrate-producing species and higher populations of pro-inflammatory genera in the fecal samples of AD patients.^80^ It should be noted, however, that while some SCFAs, like butyrate, demonstrated anti-inflammatory effects, others, such as acetate, showed pro-inflammatory effects that were concentration-dependent and influenced by the metabolic state of the host.^107^

Aging is another crucial risk factor that results in a reduction of beneficial bacteria in the gut, such as Bifidobacteria and Lactobacilli, along with an increase in pro-inflammatory commensal microbes like Enterobacteriaceae and Clostridia.^108^ This shift contributes to gut dysbiosis related to aging. Aging-related gut dysbiosis can also increase intestinal and BBB permeability, promoting neuroinflammation and neurodegeneration.^109,110^ Additionally, intestinal barriers become leaky in an inflammatory environment, resulting in a harmful positive feedback loop that promotes the entry of more microbes and their metabolites into the systemic circulation. The amyloids and LPS secreted by gut microbes^101,111^ also contribute to AD pathogenesis by inducing Aβ misfolding and aggregation.^82^ Moreover, gut microbiota alterations are associated with systemic inflammation, potentially exacerbating AD progression.^112^

Oxidative stress, which results from an imbalance between reactive oxygen species (ROS) production and antioxidant defenses, is also closely associated with aging and age-related diseases.^113^ The oxidative stress theory of aging suggests that the decline in function associated with aging results from the accumulation of damage caused by ROS.^114^ This damage impacts various cellular components, including DNA, lipids, and proteins, which can ultimately lead to mitochondrial dysfunction, telomere shortening, and cellular senescence.^115^ Age-related oxidative stress and gut dysbiosis have a bidirectional relationship, resulting in a potentially vicious cycle in various diseases. Gut dysbiosis enhances oxidative stress, leading to inflammation and immune system activation.^116,117^ Conversely, oxidative stress triggers rapid shifts in gut microbiota composition, potentially causing dysbiosis.^118^ This interplay is associated with various chronic diseases, including AD, cardiovascular disorders, and inflammatory skin conditions.^116,117,119^ Additionally, oxidative stress was also found to be a crucial pathologic mechanism that enhanced the deposition of Aβ and contributed to neuronal and synaptic loss.^120^

## 7. Probiotics and dietary interventions

The interconnectedness of the MGBA and CNS health was further reinforced with research on probiotics and dietary interventions as promising strategies to improve mental health and treatment outcomes. Probiotics have been shown to influence brain activation patterns and the gut microbiome composition in healthy adults, affecting emotional processing and memory.^121^ In this landmark study, 45 participants were divided into probiotic, placebo, and treatment groups, and their baseline functional MRI profiles were identified based on emotional decision-making and emotional recognition memory tasks.^121^ The gut microbiome from stool samples was characterized before and after a 4-week administration of probiotics. The results revealed that alterations in the gut microbiome composition were associated with changes in self-reported behavior and memory performance.^121^ *Lactobacillus plantarum* 299 v was found to enhance cognitive function and reduce kynurenine levels, a metabolite derived from tryptophan, in patients with major depressive disorder who were being treated with selective serotonin reuptake inhibitors (SSRIs).^122^ Animal studies have also revealed that chronic stress can induce lasting changes in the gut microbiota. Moreover, low-intensity repetitive transcranial magnetic stimulation (rTMS) may counteract some of these effects and reduce inflammatory processes.^123^

A recent systematic review, which screened and selected 22 studies on the preventive role of probiotics against Alzheimer’s disease (AD) using a Boolean approach, highlighted promising findings. The analysis revealed that probiotics may help slow AD progression by modulating inflammation, reducing oxidative stress, and restoring gut microbiota balance, offering a potential pathway for therapeutic intervention.^124^ The animal studies mentioned in that review are summarized in Table 2.

**Table 2.** The impact of probiotics on AD in live animal studies.

Key Strains/ Components and Interventions	Animal	Duration	Observed Effects	References
*Lactobacillus plantarum* MTCC 1325	3-month-old albino rats (Wistar strain)	60 days	Improved spatial memory increased acetylcholine levels reduced inflammatory cytokines, amyloid plaques, and NFTs in AD-induced rats	Mallikarjuna Nimgampalle et al.^125^
*Lactobacillus spp., Bifidobacterium spp.*	Adult normal-reared male Wistar rats	56 days	Restored synaptic plasticity improved cognitive functions in Aβ-injected rats	Rezaei Asl et al.^126^
*Lactobacillus reuteri, Lactobacillus rhamnosus, Bifidobacterium infantis*	Male Wistar rats	70 days	Reduction of Aβ deposition reduced inflammation markers in AD-induced rats	Mehrabadi et al.^127^
*L. acidophilus, L. fermentum, B. lactis*, and *B. longum*	Male Wistar rats	56 days	Significant improvement of spatial memory increased malondialdehyde levels, and SOD activity	Azm et al.^128^
SLAB51 (*S. thermophilus, Bifidobacterium spp.*, and *Lactobacillus spp*.)	8-week-old male 3xTg-AD mice	16 weeks (2017) & 56 weeks (2018)	Increased GLUT3 and GLUT1 levels in the hippocampal CA1 region reduced p-tau levels in the brain; increased HbA1c and IGF-IRβ in the plasma and brain Decreased p53; increased RARβ, GST, GPx, SOD, and CAT in brain homogenates	Bonfili et al.^129, 130^
VSL#3 (*Lactobacillus spp*. and *Bifidobacterium spp*.)	6–8 month-old female App^NL-G-F^ and C57BL/6 WT mice	8 weeks	Increased hippocampal SCFA acetate, butyrate, and lactate levels increased serum propionate, isobutyrate, and c-fos immunoreactivity in the brain (neuronal activity marker) reduced anxiety-like behavior	Kaur et al.^131^
*C. butyricum* (WZMC1016)	6-month-old APPswe/PS1dE9 transgenic AD mouse model and C57BL/6 WT mice	4 weeks	Improved spatial learning and memory increased fecal butyrate levels suppressed microglial activation reduced FJC-positive cells in the cortex and hippocampus reduced Aβ, Aβ42, IL-1β, α-TNF, COX-2, and p-p65 expression in brain tissue	Sun et al.^132^
*Bifidobacterium longum, L. acidophilus* lysates, vitamins, omega-3s with interval running	APP/PS1^TG^ mice	20 weeks	Increased exploratory activity, microglia, 8-oxo-guanine DNA glycosylase (OGG1) levels, *L. reuteri* populations, and cognitive performance compared to controls	Abraham et al.^133^
*Bifidobacterium longum, L. acidophilus* lysates, vitamins, omega-3s with interval running	APP/PS1^TG^ mice	20 weeks	Increased hippocampal SOD and 8-oxo-dG levels, and liver nuclear factor erythroid 2-related factor 2 (NRF-2)	Téglás et al.^134^

Recent studies with transgenic mouse models have shown that probiotics significantly improve cognitive performance, reduce AD pathology, and modulate the MGBA. These findings highlighted the potential of probiotics as a therapeutic strategy to alleviate neuroinflammation, oxidative stress, and cognitive decline associated with AD. While these promising results provided valuable insights, the complexity of human AD necessitated clinical trials to confirm the efficacy and safety of probiotics in human patients.^124^

In clinical trials, probiotics were found to potentially influence AD patients’ cognitive and metabolic health. Clinical trials are scant, and their details are summarized in Table 3.

**Table 3.** Effects of probiotics in clinical trials.

Supplement	Duration	Cohort	Observed Effects	Reference
Probiotic-enriched milk *(L. acidophilus, L. casei, L. fermentum, and B. bifidum*)	12 weeks	30 AD patients	Improved MMSE score Reduced c-reactive protein high-sensitivity, triglyceride, and MDA in serum No effect on total antioxidant capacity	Akbari et al.^135^
3 x 10^9^ CFU of various probiotics, including *L. fermentum* and *B. longum*	12 weeks	25 AD patients	Increased TYM score, cognitive function, and serum GSH Decreased 8-OHdg in serum No effect on total antioxidant capacity	Agahi et al.^136^
*Lactobacillus casei* W56 and *Bifidobacterium bifidum* W23	28 days	20 AD patients	Decreased fecal zonulin Increased *Faecalibacterium prausnitzii* and serum kynurenine levels Significant correlation in neopterin delta values and kynurenine to tryptophan ratios	Friedrich et al.^137^
*Lactobacillus acidophilus, Bifidobacterium bifidum*, and *Bifidobacterium longum* with selenium	12 weeks	79 AD patients	Positive correlation between probiotics supplements and antioxidant capacity	Tamtaji et al.^138^

Tamtaji et al. revealed a positive correlation between probiotic supplements and antioxidant capacity.^138^ Still, they contradicted the findings of Akbari and Agahi, who reported no effects at all, though the same bacterial species were used.^135,136^ Recent clinical studies on probiotic supplementation to alleviate the impact of AD showed promising results, consistent with the findings from animal models. Despite progress, challenges persist due to a lack of studies and outcome variations, highlighting the need for more comprehensive trials to understand the therapeutic potential and mechanisms of these interventions fully.

Meanwhile, clinical studies on anxiety symptoms have shown mixed results. A systematic review of probiotic and non-probiotic interventions, in the form of dietary changes, to treat anxiety disorders was performed.^139^ More than half of the studies reviewed indicated that regulating gut microbiota can positively impact anxiety symptoms. However, non-probiotic approaches have shown increased efficacy compared to probiotic approaches.^139^ The ineffectiveness of probiotic interventions in some studies may be attributed to the use of only one or two kinds of probiotics, which is limited, considering that more strains may be required to leverage their synergistic effects on gut and brain health. Additionally, dietary modifications provide a broader array of nutrients, fibers, and bioactive compounds that more effectively enhance microbiota diversity than the limited impact of single- or dual-strain probiotics.^139^ A report on the 10^th^ International Yakult Symposium held in Milan listed several challenges that need to be considered when designing clinical trials in probiotics:^140^

- **Inter-individual variation** poses unique challenges, as nutritional interventions often include subtle differences that distinct physiological responses in individuals can easily overshadow.
- **External validity** can be limited by the heterogeneity of study populations and individual variations. The findings in randomized clinical trials must be applicable to a defined population.
- **Independence of Effects**. The mixture of different species and strains in probiotic trials increases the likelihood of obtaining ambiguous results. This is compounded by systematic reviews and meta-analyses that pool studies that used different strains. This requires understanding the specific mode of action to identify the correct outcome variable and patient group.
- **Adequate characterization**. Probiotics are living organisms that evolve once they reach the recipient’s colon, depending on the substrates provided by the diet. This means that the same product may not consistently deliver the same outcome to every recipient.
- **Responder vs non-responder**. Advancements in machine learning could identify potential responders by analyzing health, genetic, and dietary data for targeted probiotic interventions.
- **Surrogate biomarkers** that show short-term changes predictive of health effects may be considered to reduce the influence of other lifestyle factors on medium-term disease markers.

Nevertheless, many unknown metabolites, with many undiscovered microbial strains, are likely to be studied as potential therapeutic interventions. More specific microbial strains and their metabolites that can modulate the GBA have been identified. For example, another strain of *L. plantarum* (IS-10506) was shown to stimulate the GBA by increasing brain-derived neurotrophic factor (BDNF), neurotrophin (NT), and serotonin (5-HTT) levels in the brain, as well as increasing intestinal serotonin (5-HT) levels.^141^ These results suggested that *L. plantarum* IS-10506 could enhance brain development and function through its effects on the GBA.^141^ A study found that alone or with exercise, probiotic intervention can delay inflammation, reduce oxidative stress, and decrease Aβ levels, thus improving cognitive function in mice.^142^

### 7.1. Neurological impact of diet and fasting

**Figure 4.**
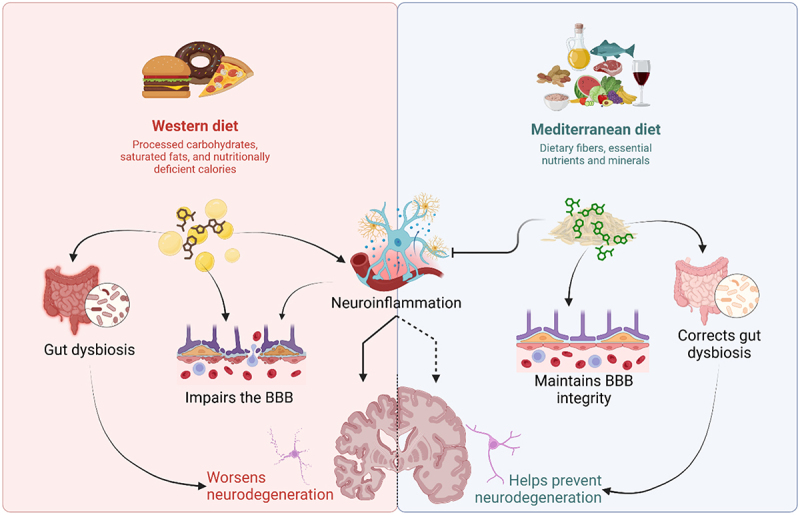
Neurological impact of different diets. Western diets (left) were primarily composed of processed carbohydrates and saturated fats that may impair the BBB and increase the likelihood of neuroinflammation and gut dysbiosis, leading to worsening of neurodegeneration. In contrast, the Mediterranean diet (right) primarily consists of essential fibers, nutrients, and minerals that help maintain the integrity of the BBB and restore gut dysbiosis, thereby preventing neuroinflammation that leads to neurological impairment—created with BioRender.com.

Although several mouse studies have shown that sucrose- and fat-enriched diets can induce depression and anxiety-like behavior,^41,143–145^ human studies are still limited. However, emerging evidence from longitudinal studies of dietary interventions, such as Mediterranean diets, has shown their potential to prevent AD by targeting neuroinflammation, altering the intestinal and blood–brain barriers, and correcting gut dysbiosis.^146^ In contrast, Western diets worsen AD progression through genetic alterations, impaired barrier function, and chronic neuroinflammation.^146^ This is likely attributed to processed foods, sugary drinks, and foods deficient in essential nutrients, fibers, and minerals, which are critical in mitigating chronic metabolic inflammation (Figure 4).^147^ Dietary patterns predominantly consisting of processed carbohydrates, saturated fats, and nutritionally deficient calories also exacerbate inflammatory conditions that promote AD.^148^ However, much more evidence is needed to establish the longitudinal effectiveness of different dietary patterns in reversing neurodegeneration in its early stages. Moreover, the relative sequence and timing of these nutritional interventions in the complex neurophysiological cascade must be investigated.^146^

Intermittent fasting (IF) is a dietary approach that involves fully or partially refraining from food during designated time intervals. In recent years, IF has garnered significant attention and interest in research.^149^ IF has been shown to activate key signaling pathways in cellular energy regulation and metabolic health, such as the AMP-activated protein kinase (AMPK) and sirtuin 1 (SIRT1) pathways. AMPK was revealed to be associated with acetyl-CoA carboxylase (ACC), when it was reported that a partially purified preparation of ACC was inactivated in a time-dependent manner upon incubation with ATP.^150^ At the time, an unknown regulatory enzyme was observed to phosphorylate ACC in response to changes in cellular energy levels.^150^ AMPK was also known to enhance metabolic health through the breakdown of available fatty acids for energy and improve the cellular uptake of glucose to support further energy production.^151,152^ SIRT1, on the other hand, was revealed to regulate the expression of metabolic genes through deacetylation, enhancing fatty acid oxidation and mitochondrial function.^153^ It was also known to promote mitochondrial biogenesis and function,^151,153,154^ promote autophagy to clear cellular debris,^155,156^ and work with AMPK to enhance fatty acid oxidative metabolism to maximize the use of cellular energy.^151,157^

Another critical mechanism underlying the neurological benefits of IF is its impact on leptin, a hormone predominantly produced by adipose tissue. Leptin plays a pivotal role in energy homeostasis and appetite regulation. During fasting, leptin levels typically decrease, which may enhance the brain’s sensitivity to leptin signals and improve metabolic efficiency. This hormonal modulation is also linked to the AMPK and SIRT1 pathways, activated during energy deficits, promoting neuronal resilience and reducing neuroinflammation. Emerging evidence suggests that leptin’s influence on hypothalamic function could further mediate fasting-induced improvements in cognitive performance and synaptic plasticity,^158^ providing a complementary pathway to the gut–brain axis interventions already discussed.

Improving mitochondrial function, reducing oxidative stress through fatty acid oxidation and activation of antioxidants, autophagy, and regulation of neuroinflammation are key processes that protect neurons and minimize stressful events that could further propagate AD. Studies in AD mouse models demonstrated that IF improved spatial memory, reduced Aβ accumulation, and suppressed neuroinflammation.^159,160^ The beneficial effects of IF were mediated by reshaping the gut microbiota, particularly enriching probiotics like Lactobacillus and modifying gut metabolites.^159,160^ Metabolomic analysis showed IF-induced alterations in carbohydrate metabolism and increased levels of specific amino acids like sarcosine and dimethylglycine, both of which can help improve cognitive decline and reduce AD pathology.^160^ Indole-3-propionic acid (IPA), a microbial tryptophan metabolite, was found to be enhanced in the serum of APP/PS1 mice following 16 weeks of intermittent fasting.^159^ This agreed with another study by the same group where IPA and SCFAs were similarly elevated in type 2 diabetic mice that underwent 28-day IF.^161^ Gamma-tocopherol and N-acetylmannosamine were essential metabolites that increased AD mice serum following IF.^159^

Regarding restoring leaky barriers in the GI wall, the results were mixed and limited to animal models. In fruit flies, brief periods of IF during early development enhanced barrier function and diminished age-related diseases.^162^ The rapid restoration of gut epithelial barriers in simian immunodeficiency virus (SIV)-infected rhesus macaques was similarly observed following probiotic administration, mediated by peroxisome proliferator-activated receptors (PPAR)α activation and mitochondrial restoration.^163^ However, gerbils that underwent long-term IF were found to have increased hippocampal IL-13, but IF did not protect the BBB or neurons from ischemia-reperfusion injury.^164^ Studies that explored the restorative role of IF in animal models of AD were limited. Still, some studies focused on the effects of IF on experimentally induced chronic cerebral hypoperfusion (CCH) in rodents. A 4-month IF regimen on male C57BL/6NTac mice was found to reduce CCH-induced neurovascular pathologies, particularly reduction in the number of leaky microvessels, degree of BBB breakdown, and loss of tight junctional proteins.^165^ IF also lessened the severity of white matter lesions, upheld myelin basic protein levels, and lowered neuronal loss in the hippocampus.^165^ Matrix metalloproteinase (MMP)-2 levels involved in the breakdown of the extracellular matrix were also found to be mitigated along with its upstream activator MT1-MMP.^165^

In a different study, the same intermittent fasting (IF) regimen enhanced cognitive learning abilities and memory while alleviating neuropathological changes, leading to a reduction in white matter lesions and neuronal loss in chronic cerebral hypoperfusion (CCH) mouse models of vascular dementia.^166^ Alterations in DNA methylation patterns in the cortical region of control subjects were restructured, and indications of reversal were noted in fasting mice at all observed time points.^166^ Another study showed that the same IF regimen on CCH mice decreased inflammasome activation and reduced the initiation of apoptotic and pyroptotic cell death pathways.^167^ This was based on the 20–30% reduction in expression of key effector proteins and cell death markers in the cerebellum.^167^

The impact of IF on the clearance of cellular deposits, particularly Aβ, was also explored. In one study, the potential of IF to restore aquaporin-4 polarity in the glymphatic pathway was revealed using APP/PS1 double transgenic mice and may significantly improve Aβ clearance from the brain.^168^ However, the limited samples allowed for immunohistochemical analysis of Aβ and should be confirmed by quantitative assays like ELISA.^168^ Also, the mechanism to explain the absence of IF effects on the wild-type controls needs to be investigated further.^168^

### 7.2. On pre, pro-, and synbiotics

Prebiotics are selectively fermented ingredients that change the GI microflora to benefit the host’s health and well-being.^169^ They are typically nondigestible oligosaccharides that serve as food for beneficial gut bacteria, promoting their growth and activity.^169^ Prebiotics have little interaction with the host but were observed to establish a balanced commensal microbiota, which can produce beneficial metabolites such as SCFAs.^170^

Probiotics are viable microorganisms that, when present in sufficient amounts, can exert positive health effects in the GI tract.^169^ Common strains in probiotic foods include lactobacilli, bifidobacteria, enterococci, and streptococci.^169^ Probiotics are known for improving GI health, and many studies have explored their potential benefits in various psychiatric disorders.^171^

Prebiotics and probiotics are older concepts, and emerging evidence suggests that synergistic combinations (synbiotics) of the two provide greater benefits.^169^ The principal idea is that prebiotics supply the essential nutrients for probiotics to flourish, increasing their positive effects. Synbiotics seek to improve the bioavailability and effectiveness of both elements, potentially providing more significant benefits than prebiotics or probiotics alone.^170^

No evidence conclusively indicates that one is more beneficial than the others. Each had shown potential benefits in different contexts and conditions, but the results were mixed and often depended on the specific application and individual patient characteristics. Probiotics have shown promise in treating major depressive disorder and AD.^170,172^ However, their efficacy may vary, and some studies have not reported significant benefits.^169^ Prebiotics had been less frequently studied in isolation for psychiatric disorders, and the evidence supporting their use was not as robust as that for probiotics. Some studies suggest that they can help establish a balanced gut microbiota, which may have downstream benefits for mental health.^170^ Finally, some studies have indicated that synbiotics increase the bioavailability of pre/probiotics. Synbiotics have shown the potential to improve cognitive function and reduce inflammation in AD; however, many finalized trials were short-term and prevented the observation of long-term effects.^169^ The cohort sizes involved were also small and diverse, limiting the generalizability of the findings.^169^

The relative advantages of prebiotics, probiotics, and synbiotics remain unclear. These options should be selected based on the specific condition being addressed and individual patient characteristics. Additional research is needed to reach more definitive conclusions.

## 8. Future directions

Several gaps still exist in the current framework encapsulating the effects of dietary interventions on gut microbiota and neurodegenerative disorders. More extensive, well-designed longitudinal studies that account for confounders such as complex diets and psychotropic medications are warranted.^173^ These findings should be able to establish causal relationships and provide a better description of the dynamic interactions between gut bacteria and brain disorders. The functional potential is just as essential to characterize as the composition of microbial communities.^174,175^ The specific mechanisms by which gut bacteria influence brain function and behavior can then be elucidated. Other mechanistic details on how bacteria affect the CNS, including the roles of neurotransmitters, the hypothalamic‒pituitary‒adrenal axis, and inflammation, should also be considered.^175,176^

While some exploratory clinical trials investigated interventions to treat brain disorders by targeting the MGBA, more rigorous clinical trials on prebiotics, probiotics, and fecal microbiota transplantation focusing on well-defined populations and standardized intervention protocols are warranted.^177,178^ More studies should also explore the influence of diet on modulating the gut microbial composition and resulting mental health outcomes.^175^ Studies should also encompass diverse populations, varying in age, and consider other comorbidities such as cancer and approaches to cancer treatment , to better understand the broader relevance of findings related to the gut microbiota and mental health.^179^

Advancements in modern biotechnology, including several new approaches, allow for faster and more accurate data mining, enabling researchers to explore these questions more thoroughly and gain deeper insights. Metagenomics, metatranscriptomics, and metabolomics should be expanded to understand better the gut microbiota’s biological properties and their impact on host physiology.^175^

## 9. Conclusion

Exploring the GBA has revealed a dynamic communication network between the gastrointestinal tract and the central nervous system. This review underscores the impact of gut microbiota diversity, neuromodulators, and hormones on CNS health and the propensity to develop AD. Via a combination of diversity and functionality, microbial fitness plays a pivotal role in understanding the intricate relationship between gut health and cognitive fitness. The gut–brain axis (GBA) serves as a conduit through which the gut microbiota influences brain function, demonstrating that disruptions in one system can reflect and amplify dysfunctions in the other. This bidirectional interaction between the gut and brain underscores the importance of microbial diversity and functionality in maintaining cognitive health.

Gut microbes act as tiny conductors, orchestrating the symphony of neuromodulators, including serotonin, dopamine, and short-chain fatty acids (SCFAs). These metabolites directly impact brain health by supporting synaptic plasticity, neuronal energy metabolism, and neuroinflammatory modulation. Dysbiosis, marked by a loss of microbial fitness, undermines these processes, contributing to neurodegenerative diseases such as AD.

Restoring microbial diversity offers a promising avenue for mitigating cognitive decline. A potential solution to decline is ongoing research on probiotic interventions that restore gut homeostasis and decrease the likelihood of inflammatory events contributing to neurodegeneration. Probiotics, dietary interventions, and microbial metabolites have shown the potential to alter brain function and behavior, suggesting new strategies for managing conditions such as depression, anxiety, and neurodegenerative diseases. Despite these advancements, many unknowns remain regarding the specific microbial strains and metabolites involved in GBA signaling. Future research should focus on elucidating these details to develop targeted microbiome-based therapies. The discovery of new microbial metabolites and their roles in GBA interactions will likely expand our therapeutic toolkit, potentially revolutionizing our approach to treating AD and other neurodegenerative diseases.

## Data Availability

This is a review article; no new datasets were generated or analyzed. All data discussed in this paper are publicly available and appropriately cited.

## Appendix: Table A1

**Table:**

QSM	Molecular Type	Chemical structure	Type of bacteria	Mechanism of action	Biological Functions	Host interactions
N-acyl-homoserine lactones (AHLs)	Neutral lipid	Homoserine lactone ring with an acyl side chain^1^	Gram-negative	Binding to LuxR receptors, activating transcription^2^	Biofilm formation, virulence factor production^3,4^	Interaction with host immune cells, modulation of immune response.^5^
Oligopeptides	Protein	Short peptides consisting of 5–20 amino acid residues^6^	Gram-positive	Binding to two-component systems; Rap, NprR, PlcR, and PrgX^7^	Regulation of gene expression, biofilm formation, competence, sporulation, virulence factor production^8–11^	Penetrate the BBB and influence neurological disorders^12^
Autoinducer-2	Furanosyl borate diester^7^	Furanose linked to borate through ester bonds^7^	Both Gram-negative and Gram-positive^7^	Binding to luxS system reported in several bacteria (i.e., bacteriocin production)^7^	Interspecies communication, luminescence, biofilm formation^7^ Regulation of virulence factors^13^	Influences plant growth and resistance to phytopathogens^14^ Increase disease pathogenicity (i.e., acute pneumonia)^14^
Autoinducer-3	Derivative of threonine dehydrogenase (Tdh) and “abortive” tRNA synthetase reactions^15^	Dimerization of two aminoacetone molecules followed by oxidation^16^	Both Gram-negative and Gram-positive^15^	Binds to QseC sensor kinase^17^	Bacterial communication, virulence regulation, particularly in EHEC^17, 18^	Responds to host epinephrine and norepinephrine^17^
Pseudomonas Quinolone Signal (PQS)	Quinoline derivative^19^	4-quinolone core hydroxylated at the 3-position and a heptyl alkyl chain at the 2-position^20^	Gram-negative	Binds to the PqsR system^21^	Regulates virulence factor expression, mediates iron acquisition, induces cytotoxicity, facilitates outer-membrane vesicle biogenesis^22, 23^	Modulates host immune responses^22^
Indoles	Aromatic heterocyclic organic compound	Bicyclic compound with a benzene ring fused to a pyrrole ring	Both Gram-negative and Gram-positive^24^	Interferes with the folding of QS regulators^25^	Controls biofilm production,^25,26^ increased expression of antibiotic-resistance genes^27^	Maintains intestinal homeostasis and modulates immune responses^28^
Short-chain fatty acids (SCFAs)	Fatty acid	Carboxylic acid with an aliphatic chain	Both Gram-negative and Gram-positive (predominantly)	Interfere with QS; Decrease expression of QS regulators (i.e., abaR)^29^	Repress virulence; interfere with biofilm production^30^ Affect epigenetic regulation of genes in metabolic disorders^31^ Structural role in QSM^32^ Membrane perturbation^33^	Modulate microglial inflammation in the brain^34^

### References

1. Li, Z. and S.K. Nair, *Quorum
  sensing: how bacteria can coordinate activity and synchronize their response to
  external signals?* Protein Sci, 2012. 21(10):
  p. 1403–17.
2. Fuqua, W.C., S.C.
  Winans, and E.P. Greenberg, *Quorum
  sensing in bacteria: the LuxR-LuxI family of cell density-responsive
  transcriptional regulators*. J Bacteriol, 1994. 176(2): p. 269–75.
3. Kumar, L., et al., *Molecular Mechanisms and Applications of
  N-Acyl Homoserine Lactone-Mediated Quorum Sensing in Bacteria*. Molecules,
  2022. 27(21).
4. Ma, H., et al., *The biological role of N-acyl-homoserine
  lactone-based quorum sensing (QS) in EPS production and microbial community
  assembly during anaerobic granulation process*. Scientific Reports, 2018. 8(1): p. 15793.
5. Xiao, Y., et al., *Impact of quorum sensing signaling molecules
  in gram-negative bacteria on host cells: current understanding and future
  perspectives*. Gut Microbes, 2022. 14(1):
  p. 2039048.
6. Monnet, V. and R.
  Gardan, *Quorum-sensing regulators in
  Gram-positive bacteria: ’cherchez le peptide’*. Mol Microbiol, 2015. 97(2): p. 181–4.
7. Dicks, L.M.T., *How does Quorum Sensing of Intestinal
  Bacteria Affect Our Health and Mental Status?* Microorganisms, 2022. 10(10): p. 1969.
8. Aggarwal, S., et al.,
  *The leaderless communication peptide
  (LCP) class of quorum-sensing peptides is broadly distributed among Firmicutes*.
  Nature Communications, 2023. 14(1):
  p. 5947.
9. McBrayer, D.N., C.D.
  Cameron, and Y. Tal-Gan, *Development and
  utilization of peptide-based quorum sensing modulators in Gram-positive
  bacteria*. Org Biomol Chem, 2020. 18(37):
  p. 7273–7290.
10. Sahreen, S., et al. *Exploring the Function of Quorum Sensing
  Regulated Biofilms in Biological Wastewater Treatment: A Review*.
  International Journal of Molecular Sciences, 2022. 23,  DOI:
  10.3390/ijms23179751.
11. Wynendaele, E., et
  al., *Quorum Sensing Peptides Selectively
  Penetrate the Blood-Brain Barrier*. PLOS ONE, 2015. 10(11): p. e0142071.
12. Wynendaele, E., et
  al., *Quorumpeps database: chemical space,
  microbial origin and functionality of quorum sensing peptides*. Nucleic
  Acids Research, 2012. 41: p. D655–
  D659.
13. Vendeville, A., et
  al., *Making ’sense’ of metabolism:
  autoinducer-2, LUXS and pathogenic bacteria*. Nature Reviews Microbiology,
  2005. 3(5): p. 383–396.
14. Majdura, J., et al., *The Role of Quorum Sensing Molecules in
  Bacterial-Plant Interactions*. Metabolites, 2023. 13(1).
15. Kim, C.S., et al., *Characterization of Autoinducer-3 Structure
  and Biosynthesis in E. coli*. ACS Central Science, 2020. 6(2): p. 197–206.
16. Hernandez, D.E. and
  H.O. Sintim, *Quorum Sensing Autoinducer-3
  Finally Yields to Structural Elucidation*. ACS Central Science, 2020. 6(2): p. 93–96.
17. Clarke, M.B., et al., *The QseC sensor kinase: A bacterial
  adrenergic receptor*. Proceedings of the National Academy of Sciences, 2006.
  103: p. 10420–10425.
18. Walters, M.S., M.P.
  Sircili, and V. Sperandio, *AI-3 Synthesis
  Is Not Dependent on luxS in Escherichia coli*. Journal of Bacteriology,
  2006. 188: p. 5668–5681.
19. Heeb, S., et al., *Quinolones: from antibiotics to
  autoinducers*. FEMS Microbiology Reviews, 2011. 35(2): p. 247–274.
20. Li, A., J. Schertzer,
  and X. Yong, *Molecular conformation
  affects the interaction of the Pseudomonas quinolone signal with the bacterial
  outer membrane*. Journal of Biological Chemistry, 2018. 294: p. jbc.AC118.006844.
21. Pesci, E.C., et al., *Quinolone signaling in the cell-to-cell
  communication system of Pseudomonas aeruginosa*. Proceedings of the National
  Academy of Sciences of the United States of America, 1999. 96 20: p. 11229–34.
22. Lin, J., et al., *The Pseudomonas Quinolone Signal (PQS): Not
  Just for Quorum Sensing Anymore*. Front Cell Infect Microbiol, 2018. 8: p. 230.
23. Szamosvári, D., et
  al., *Beyond iron: metal-binding activity
  of the Pseudomonas quinolone signal-motif*. Organic & Biomolecular
  Chemistry, 2023. 21(25): p.
  5158–5163.
24. Han, T.H., et al., *Environmental factors affecting indole
  production in Escherichia coli*. Res Microbiol, 2011. 162(2): p. 108–16.
25. Kim, J. and W. Park, *Indole inhibits bacterial quorum sensing
  signal transmission by interfering with quorum sensing regulator folding*.
  Microbiology (Reading), 2013. 159(Pt
  12): p. 2616–2625.
26. Wu, W., et al., *Indole signaling enhances biofilm formation
  and quorum sensing in sequencing biofilm batch reactors*. Journal of
  Environmental Chemical Engineering, 2024. 12(3):
  p. 112494.
27. Chunxiao, D., et al., *Metagenomic analysis reveals indole
  signaling effect on microbial community in sequencing batch reactors: Quorum
  sensing inhibition and antibiotic resistance enrichment*. Environmental
  Research, 2023. 229: p. 115897.
28. Kumar, A. and V.
  Sperandio, *Indole Signaling at the
  Host-Microbiota-Pathogen Interface*. mBio, 2019. 10(3).
29. Nicol, M., et al., *Unsaturated Fatty Acids Affect Quorum
  Sensing Communication System and Inhibit Motility and Biofilm Formation of
  Acinetobacter baumannii*. Int J Mol Sci, 2018. 19(1).
30. Kumar, P., et al., *Fatty Acids as Antibiofilm and Antivirulence
  Agents*. Trends in Microbiology, 2020. 28(9):
  p. 753–768.
31. Remely, M., et al., *Effects of short chain fatty acid producing
  bacteria on epigenetic regulation of FFAR3 in type 2 diabetes and obesity*.
  Gene, 2014. 537 1: p. 85–92.
32. He, Y.-W., et al., *DSF-family quorum sensing signal-mediated
  intraspecies, interspecies, and inter-kingdom communication*. Trends in
  Microbiology, 2023. 31(1): p. 36–50.
33. Luo, W., et al., *Roles and Regulation of Quorum Sensing of
  Acidophiles in Bioleaching: A Review*. Microorganisms, 2024. 12(3): p. 422.
34. Churchward, M.A., et
  al., *Short-chain fatty and carboxylic
  acid changes associated with fecal microbiota transplant communally influence
  microglial inflammation*. Heliyon, 2023. 9.
